# The Ca^2+^-CaM Signaling Pathway Mediates Potassium Uptake by Regulating Reactive Oxygen Species Homeostasis in Tobacco Roots Under Low-K^+^ Stress

**DOI:** 10.3389/fpls.2021.658609

**Published:** 2021-06-07

**Authors:** Yingfeng Wang, Xiaoyan Dai, Gaoqiang Xu, Zhuoyi Dai, Peiyu Chen, Tongjin Zhang, Huifen Zhang

**Affiliations:** College of Tobacco Science, Henan Agricultural University, Zhengzhou, China

**Keywords:** calmodulin, calcium signaling, low-potassium stress, reactive oxygen species, root

## Abstract

Potassium (K^+^) deficiency severely threatens crop growth and productivity. Calcium (Ca^2+^) signaling and its sensors play a central role in the response to low-K^+^ stress. Calmodulin (CaM) is an important Ca^2+^ sensor. However, the mechanism by which Ca^2+^ signaling and CaM mediate the response of roots to low-K^+^ stress remains unclear. In this study, we found that the K^+^ concentration significantly decreased in both shoots and roots treated with Ca^2+^ channel blockers, a Ca^2+^ chelator, and CaM antagonists. Under low-K^+^ stress, reactive oxygen species (ROS) accumulated, and the activity of antioxidant enzymes, NAD kinase (NADK), and NADP phosphatase (NADPase) decreased. This indicates that antioxidant enzymes, NADK, and NADPase might be downstream target proteins in the Ca^2+^-CaM signaling pathway, which facilitates K^+^ uptake in plant roots by mediating ROS homeostasis under low-K^+^ stress. Moreover, the expression of *NtCNGC3*, *NtCNGC10*, K^+^ channel genes, and transporter genes was significantly downregulated in blocker-treated, chelator-treated, and antagonist-treated plant roots in the low K^+^ treatment, suggesting that the Ca^2+^-CaM signaling pathway may mediate K^+^ uptake by regulating the expression of these genes. Overall, this study shows that the Ca^2+^-CaM signaling pathway promotes K^+^ absorption by regulating ROS homeostasis and the expression of K^+^ uptake-related genes in plant roots under low-K^+^ stress.

## Introduction

Potassium (K^+^) is one of the most important macronutrients in the cells of higher plants and is indispensable for plant growth and development (Leigh and Wyn Jones, 1984). K^+^ is a soluble ion that is involved in many physiological processes, including plasma membrane hyperpolarization, stomatal movement, and osmotic regulation (Wang et al., 2013); it also plays an important role in the response to various abiotic stresses in plants (Hasanuzzaman et al., 2018). The soil is the primary source of K^+^ for plants. However, the supply of K^+^ is often low in soils (e.g., one-quarter of arable soils in China; two-thirds of the wheat soil in Southern Australia), and this can limit crop yield and quality, especially in the early stages of plant establishment (Rengel and Damon, 2008). As K^+^ cannot be synthesized in plant cells, K^+^ is typically acquired from the soil by in plants *via* the roots. The expression of genes for some transporters, channels, and signaling cascades in plant roots involved in uptake, transport, and transduction and distribution can be induced by K^+^ deficiency. The expression level of *AtHAK5* was most strongly and consistently upregulated during 48 h, 96 h, and 7 days of K^+^ deficiency (Gierth et al., 2005). In rice, the transcript levels of *OsHAK1*, *OsHAK5*, *OsHAK7*, and *OsHAK16* were significantly increased in the roots under K^+^ deficiency (Banuelos et al., 2002; Okada et al., 2008). Under low-K^+^ stress in *Arabidopsis thaliana*, *AtAKT1* activity is regulated through a heteromeric K^+^ channel formed by the interaction of *AtKC1* with *AtAKT1* and with *AtCIPK23* (Jeanguenin et al., 2011; Wang and Wu, 2013; Wang et al., 2016). There is, thus, a need to explore the mechanism of K^+^ absorption and its role in determining the distribution of K^+^ under low-K^+^ stress. Such work could provide insight into how the effects of soil K^+^ deficiency on plants could be alleviated.

In higher plants, several important K^+^ shortage-activated signaling cascades are activated under K^+^ deficiency. To date, reactive oxygen species (ROS; Hernandez et al., 2012), phytohormones (e.g., ethylene, auxin, and jasmonic acid; Ashley et al., 2006; Wang et al., 2012; Li et al., 2017), calcium (Ca^2+^; Behera et al., 2017), and phosphatidic acid (Shen et al., 2020) have been shown to play a role in plant K^+^ uptake under K^+^ deficiency. Ca^2+^ signaling is one of the most important signaling systems in the responses of plants to low-K^+^ stress (Wang et al., 2018). In higher plants, the intracellular Ca^2+^ concentration increases in response to various biotic and abiotic stimuli (White and Broadley, 2003). Ca^2+^ signals are then perceived, decoded, and further transduced by Ca^2+^ sensors and their targets such as calmodulin (CaM), CaM-like protein (CML), calcium-dependent protein kinase (CDPK), calcineurin B-like protein (CBL), and CBL-interacting protein kinases (CIPKs; Sanders et al., 2002). Several studies have suggested that the CBL-CIPK signaling system plays a role in decoding and translating Ca^2+^ signatures in the response to K^+^ deficiency (Luan, 2009; Lan et al., 2011; Thoday-Kennedy et al., 2015). The CBLs-CIPKs-AKT1/AKT2/HAK5 pathways have been identified to play a role in the response to low-K^+^ stress in several plant species. Several CBL/CIPK complexes have been reported to modulate the activity of the K^+^ channels and transporters such as AKT1 (Xu et al., 2006), AKT2 (Held et al., 2011), and HAK5 (Ragel et al., 2015). ROS, such as hydrogen peroxide (H_2_O_2_), superoxide radical (O^•^_2_^−^), hydroxyl radical (OH•), and singlet oxygen (^1^O_2_), are known to negatively affect cellular metabolism; however, they can also play an important role in many signal transduction pathways. H_2_O_2_ has also been shown to be involved in the response to low-K^+^ stress. H_2_O_2_ rapidly accumulates in response to K^+^ deprivation, which modulates the expression of several genes and the kinetics of K^+^ uptake (Shin and Schachtman, 2004). Kim et al. (2010) suggested that *RCI3*, a member of the type III peroxidase (POD) family, mediates the production of ROS, which affects the regulation of *AtHAK5* expression under K^+^-deprived conditions (Kim et al., 2010).

Calmodulin is one of the best characterized Ca^2+^ sensor protein. CaM has no enzymatic and catalytic activity of its own, but apo-CaM or the binding of Ca^2+^ to CaM forms a Ca^2+^-CaM complex, which can modulate various cellular processes by activating several target proteins (Kim et al., 2009). The Ca^2+^-CaM complex is involved in the interpretation of Ca^2+^ signaling in biotic and abiotic stresses, especially in response to environmental stress, by regulating the activities of several downstream target proteins. Several studies have shown that the Ca^2+^-CaM complex is involved in the responses to oxidative stress, phytohormones, osmotic stress, salt and drought stress, heavy metal, heat shock, and chilling (Snedden and Fromm, 2001; White and Broadley, 2003; Zeng et al., 2015). However, little is known about CaM interactions during K^+^ uptake in plants, including the regulation of ion channels and transporters. Kurosaki et al. (1994) documented the presence of a plant cyclic nucleotide-gated Ca^2+^ channel that is activated by cAMP and negatively regulated by CaM (Kurosaki et al., 1994). Increases in CaM blockers or anti-CaM restored the Ca^2+^ flux across the plasma membrane. Some of the inward K^+^ channels have also been shown to be regulated by cAMP (Kurosaki, 1997).

Reactive oxygen species are also important for the production of Ca^2+^ signals in plant cells under low-K^+^ stress (Demidchik and Maathuis, 2007). H_2_O_2_ in plant cells can trigger an increase in the cytosolic Ca^2+^ concentration in response to K^+^ deficiency (Shin and Schachtman, 2004; Shin et al., 2005). However, elevated Ca^2+^ levels can induce the production of H_2_O_2_, which in turn leads to the influx of Ca^2+^ (Demidchik et al., 2007). Several studies have indicated that the Ca^2+^-CaM complex is involved in the regulation of ROS homeostasis by regulating the activities of several downstream target proteins. The Ca^2+^-CaM complex can reduce H_2_O_2_ levels in plants by binding to plant catalase (CAT) and enhancing its catalytic activity (Yang and Poovaiah, 2002). CAT is the major H_2_O_2_ scavenger enzyme in higher plants, and it can catalyze the degradation of H_2_O_2_ into water and oxygen. CaM in maize can also regulate superoxide dismutase (SOD), another class of ROS-scavenging enzymes (Gong and Li, 1995). NAD kinase (NADK) is the only enzyme known to synthesize NADP (including NADP^+^ and NADPH) by phosphorylating NAD (including NAD^+^ and NADH; Tai et al., 2019). Some studies have shown that a proper NADP(H)/NAD(H) ratio is necessary for responding to both abiotic and biotic stresses in different plants (Hashida et al., 2009; Li et al., 2018; Tai et al., 2019). NADK also plays an important role in the ROS scavenging system by regulating the balance of NADP(H)/NAD(H) (Li et al., 2018). NADPH can reactivate CAT to promote antioxidative activity (Kirkman and Gaetani, 1984). Several studies have shown that CaMs, the unique activators of NADK, play a key role in the tolerance of plants to various stresses by activating NADK to mediate the NADP(H)/NAD(H) balance, and this activation is Ca^2+^-dependent (Delumeau et al., 2000; Ruiz et al., 2002; Zeng et al., 2015). NADK-deficient mutants are sensitive to oxidative stress. When oxidative stress is caused by UVB, heat shock, drought, or salinity, the total NADPH levels of *AtNADK2*-deficient mutant plants are lower than those of wild-type plants, indicating that the *atnak2* mutant is more sensitive to oxidative stress (Chai et al., 2005).

Plant roots are the organs most sensitive to K^+^ deficiency signals, and the absorption of K^+^ by plants mainly depends on the roots. Whether the K^+^ uptake by plant roots can be directly regulated by the Ca^2+^-CaM signaling pathway under low-K^+^ stress in plants remains unclear. The aim of this study was to elucidate the mechanisms underlying the regulation of K^+^ uptake in plant roots by Ca^2+^ and the Ca^2+^-CaM signaling pathway under low-K^+^ stress. The results of this study not only enhance our understanding of Ca^2+^ signaling pathway transduction but also provide new insights into the role of the roots in mediating the response to low-K^+^ stress.

## Materials and Methods

### Plant Materials, Growth Conditions, and Experimental Treatments

*Nicotiana tabacum* cv. K326 was the study plant. Plants were grown hydroponically in a growth room with 65–70% relative humidity, 28 ± 2°C day temperature, 18 ± 2°C night temperature, and a photoperiod of 14 h light/10 h dark. The seeds were first germinated in an I-shaped square seedling sponge with Hoagland’s solution. The germinated seeds were then transferred to vermiculite. Tobacco plant seedlings with three true leaves were transferred to hydroponic plastic pots for experiments. Plants were first transferred to K^+^-free Hoagland nutrient solution for 48 h of K^+^ starvation and then placed in modified Hoagland nutrient solutions with two K^+^ concentrations for cultivation. There were two K^+^ levels in the experiment: normal 5 mmol/L and low 0.15 mmol/L. KH_2_PO_4_ was replaced by NaH_2_PO_4_ in the Hoagland nutrient solution, the K^+^ concentration was controlled by changing the concentration of KNO_3_, and insufficient concentrations of NO_3_^−^ were replaced by NaNO_3_. The pH of the nutrient solution was adjusted to 6.5–7. Six replicates were conducted for each treatment. The nutrient solution was aerated every day and replaced once every 4 days.

**Experiment I**: Effect of Ca^2+^ signaling on K^+^ uptake in tobacco roots under low-K^+^ stress. After 8 days of the two K^+^ levels treatments, 50 μmol/L verapamil (Vp, Ca^2+^ channel blocker), 200 μmol/L lanthanum chloride (LaCl_3_, Ca^2+^ channel blocker), or 2.5 mmol/L EGTA (Ca^2+^ chelator) was added to the nutrient solutions. Samples were then taken at 4 days following treatment for index analyses. The concentrations of these chemicals were determined based on the results of previous experiments.

**Experiment II**: Effect of CaM on K^+^ uptake in tobacco roots under low-K^+^ stress. Tobacco plants were first treated with two K^+^ levels for 8 days, and then 0.1 mmol/L chlorpromazine (CPZ, CaM antagonist) or 0.1 mmol/L trifluoperazine (TFP, CaM antagonist) was added to the nutrient solution. Samples were taken after 4 days of CaM antagonist treatment, and index assays were performed. The concentrations of CPZ and TFP in the experiments were determined based on the results of previous experiments.

### Plant Biomass and Root Physiological Characteristics

Both fresh weight and dry weight were used to estimate plant biomass. First, the fresh weight of the shoot and root of the plant were measured. The dry weight was measured by oven drying each part of the plant at 105°C for 15 min and then at 80°C to a constant weight. The root soluble protein content was determined at 595 nm by the Coomassie Brilliant Blue G-250 binding method (Bradford, 1976). Root activity was determined at 485 nm by the triphenyl tetrazolium chloridemethod (Zhang et al., 2013). Colorimetry was performed using a microplate reader (Tecan, Spark 10M, Switzerland).

### K^+^ Concentration

The K^+^ of plant shoots and roots was extracted by 1 mmol/L hydrochloric acid (Xu et al., 2011), and the K^+^ concentration in the extracting solution was measured by a flame photometer (FP6400, China); standards were prepared with KCl.

### ROS Accumulation

The H_2_O_2_ and O^•^_2_^−^ content were measured using a reagent kit (BC3595 and BC1295, Solarbio, Beijing, China) as per the manufacturer’s protocol (Liu et al., 2018). In brief, the H_2_O_2_ content was measured by reacting the extracting solution with 15% NH_4_OH and 10% TiCl4 and then measuring the absorbance at 410 nm. The extracting solution of O^•^_2_^−^ was reacted with p-aminobenzenesulfonamide and N-1-naphthylethylenediamin dihydrochloride, and the absorbance of the reaction mixture was determined at 530 nm. Colorimetry was performed using a microplate reader (Tecan, Spark 10M, Switzerland).

### Antioxidant Enzyme Activities

The plant roots were ground and homogenized in phosphate buffers. After centrifugation, the enzymes were extracted from the supernatant. The enzyme extract was then reacted with 50 mmol/L phosphate buffer (pH 7.0) and 15 mmol/L H_2_O_2_. Finally, CAT activity was calculated by determining the decrease in absorbance per min at 240 nm. POD activity was measured using the guaiacol method, and SOD activity was determined by the nitro blue tetrazoliummethod, as described previously (Ma et al., 2020). Colorimetry was performed using a microplate reader (Tecan, Spark 10M, Switzerland).

### NADK and NADPase Activities

The NADK and NADP phosphatase (NADPase) activities were measured using reagent kits (Comin, Suzhou, China). Briefly, the crude extracts were obtained by grinding in specific extraction buffers. After centrifugation, the supernatants were used for enzyme activity assays. The NADK and NADPase activities were assayed by detecting changes in absorbance at 340 and 660 nm, respectively, per the protocols supplied in the respective kits. Colorimetry was performed using a microplate reader (Tecan, Spark 10M, Switzerland).

### CaM Content

The CaM content was measured using the Plant CaM ELISA Kit (Meibiao Biological Technology, Jiangsu, China). Briefly, the samples were ground and extracted in PBS buffer (pH 7.4). After centrifugation, the diluted extracting solutions and enzyme labeling reagent were added to each sample well and incubated for 1 h. After coloration, the CaM content was determined at 450 nm. Colorimetry was performed using a microplate reader (Tecan, Spark 10M, Switzerland).

### Quantitative Real-Time PCR

Extraction of total RNAs and synthesis of cDNAs were conducted following the methods of Xia et al. (2018). Gene-specific primers were used for the quantitative real-time PCR (qPCR; Supplementary Table S1). qPCR assays were conducted using SYBR Green PCR Master Mix (Tiangen Biotech, China) in 20 μl reaction mixtures on an IQ5 light cycler system (Bio-Rad, Hercules, CA, United States). The expression transcription level of each gene was calculated using to the method of 2^-ΔΔCt^ method as previously described, and *NtActin* was used as the reference gene (Xu et al., 2019).

### Statistical Analysis

Microsoft Excel (Microsoft Corporation, United States) was used for data collation, SPSS (version 17.0, SPSS Inc., Chicago, IL, United States) was used for statistical analysis, and GraphPad Prism (v 8.0.2 GraphPad Software Inc., CA, United States) was used to construct graphs. All results were expressed as average values ± SD (*n* = 3).

## Results

### Effect of Ca^2+^ Channel Blockers and a Ca^2+^ Chelator on Plant Biomass and Root Physiological Characteristics Under Low-K^+^ Stress

**Figure 1:**
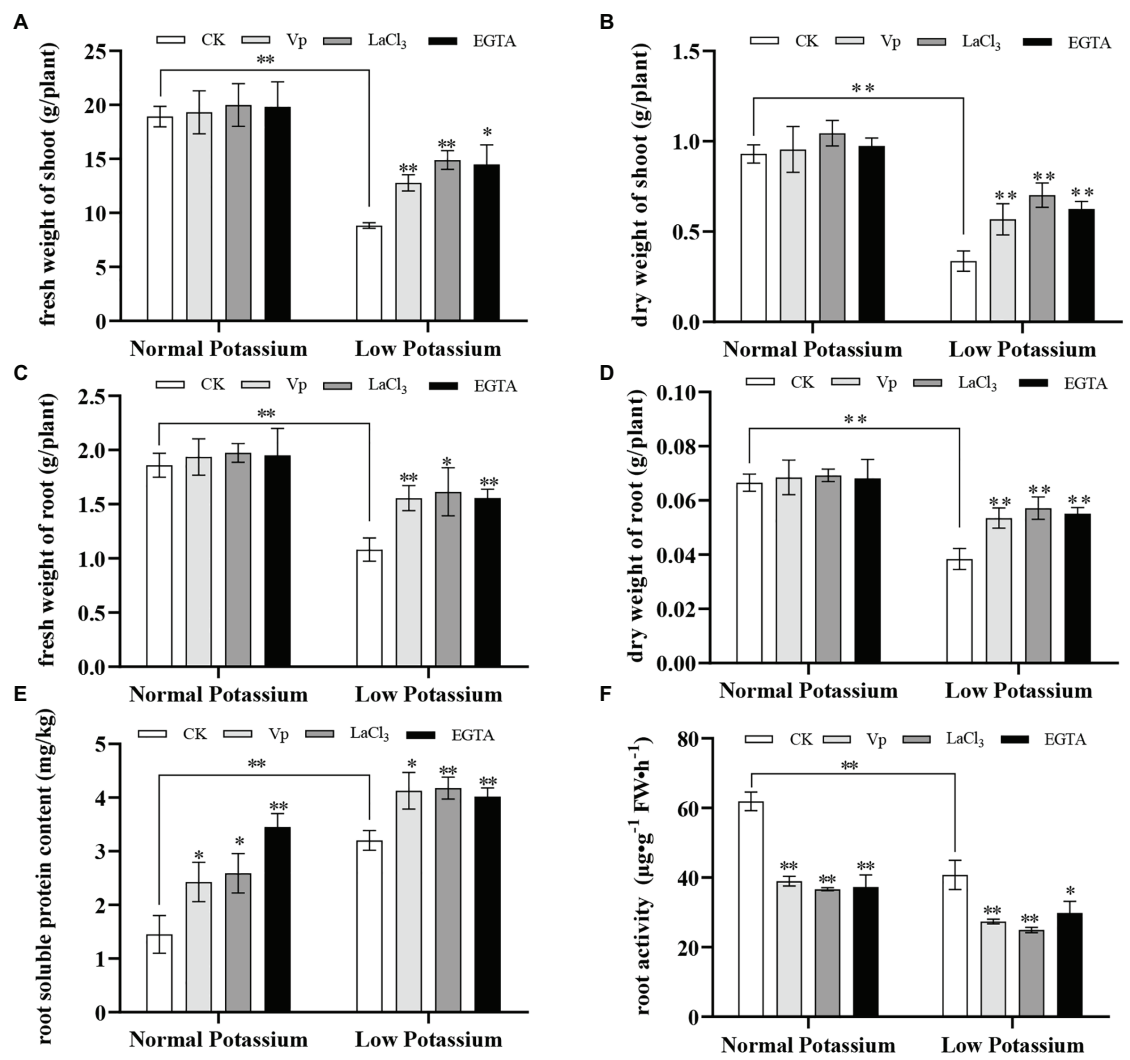
Analysis of plant biomass and root physiological characteristics under treatment with two calcium (Ca^2+^) channel blockers and a Ca^2+^ chelator at two potassium levels. Fresh and dry weights of shoots and roots **(A–D)**, root soluble protein content **(E)**, and root activity **(F)** were expressed as mean ± SD (*n* = 3). * and ** indicate significant differences compared with control (CK) at *p* < 0.05 and *p* < 0.01, respectively, by Student’s *t*-test.

Calcium signaling has often been noted in response to low-K^+^ stress, and treatments with Ca^2+^ channel blockers and Ca^2+^ chelators are often used in studies of Ca^2+^ signaling. To characterize the effects of Ca^2+^ channel blockers and a Ca^2+^ chelator on plant biomass and root physiological characteristics, we measured the fresh and dry weights of shoots and roots, root soluble protein content, and root activity in blocker-treated, chelator-treated, and control (CK) plants under different K^+^ levels. The fresh and dry weights of shoots and roots of the CK plants were significantly lower in the low K^+^ treatment than in the normal K^+^ treatment (Figures 1A–D). This suggests that low K^+^ stress could significantly inhibit the accumulation of plant biomass. In the low K^+^ treatment, the fresh and dry weights of the shoots and roots were significantly increased in blocker-treated and chelator-treated plants than in CK plants (Figures 1A–D). This indicates that inhibition of intracellular Ca^2+^ signaling may partially restore the reduction in plant biomass associated with low-K^+^ stress.

Next, we measured root physiological characteristics, including root soluble protein content and root activity. We found that the low K^+^ treatment increased root soluble protein content but decreased root activity. After adding blockers and a chelator, the root soluble protein content significantly increased in both normal and low K^+^ treatments compared with CK plants (Figure 1E). Root activity was significantly lower in the blocker-treated and chelator-treated plants compared with CK plants (Figure 1F). This indicates that interfering with Ca^2+^ transport under low-K^+^ stress can affect root activity and the root soluble protein content. These results also indicated that the concentrations of the two Ca^2+^ blockers and the Ca^2+^ chelator selected in the experiment had similar effects on plant roots.

### Treatment With Ca^2+^ Channel Blockers and a Ca^2+^ Chelator Reduces the K^+^ Concentration in Various Parts of Tobacco Plants Under Low-K^+^ Stress

**Figure 2:**
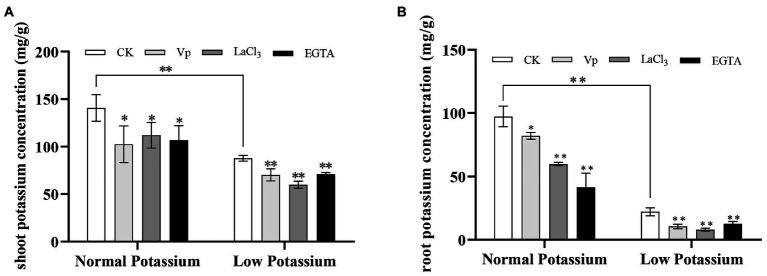
Effect of two Ca^2+^ channel blockers and a Ca^2+^ chelator on the potassium concentration in the shoots **(A)** and roots **(B)** of tobacco plants. ^*^ and ^**^ indicate significant differences between CK and treated plants at *p* < 0.05 and *p* < 0.01, respectively, by Student’s *t*-test (*n* = 4).

To explore the effect of the Ca^2+^ signaling pathway on the K^+^ concentration in tobacco plants, we assessed the K^+^ concentration in tobacco shoots and roots in the normal and low K^+^ treatments following the addition of Ca^2+^ channel blockers and a Ca^2+^ chelator. The K^+^ concentration of the shoots and roots was much higher in the normal K^+^ treatment than in the low K^+^ treatment (Figure 2). The shoot and root K^+^ concentration of the CK plants decreased by 60 and 340%, respectively, after low K^+^ treatment. In the low K^+^ treatment, the K^+^ concentration of the shoots and roots was significantly lower in the three treated plants than in CK plants (Figure 2). For example, the root K^+^ concentration of the blocker-treated and chelator-treated plants was reduced by an average of 53% relative to CK plants in the low K^+^ treatment. Similar results were obtained for the normal K^+^ treatment. These blockers and the chelator significantly suppressed the K^+^ concentration of plants in the normal and low K^+^ treatments. This indicates that interference of Ca^2+^ transport can affect the accumulation of K^+^ in plants and that that Ca^2+^ signaling is important for plant K^+^ uptake.

### Treatment With the Ca^2+^ Channel Blockers and a Ca^2+^ Chelator Increases the Accumulation of ROS in Tobacco Plant Roots Under Low-K^+^ Stress

**Figure 3:**
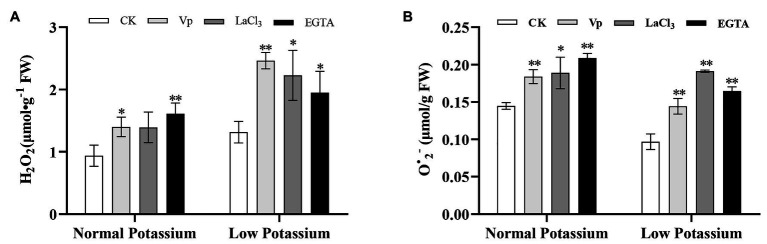
Effect of two Ca^2+^ channel blockers and a Ca^2+^ chelator on H_2_O_2_ **(A)** and O^•^_2_^−^ **(B)** in tobacco roots. * and ** indicate significant differences between CK and treated plants at *p* < 0.05 and *p* < 0.01, respectively, by Student’s *t*-test.

To determine whether ROS are involved in Ca^2+^ signaling-mediated K^+^ uptake in plants under low-K^+^ stress, the levels of H_2_O_2_ and O^•^_2_^−^ in tobacco roots were measured. The H_2_O_2_ and O^•^_2_^−^ levels were significantly higher in the roots of the blocker-treated and chelator-treated plants than in CK plants (Figure 3). After blocking Ca^2+^ signaling, the accumulation of ROS was significantly increased in the roots in the low and normal K^+^ treatments. The H_2_O_2_ content was higher in CK, blocker-treated, and chelator-treated plants after low K^+^ treatments than after normal K^+^ treatments (Figure 3A). These results indicated that interference with Ca^2+^ transport leads to the accumulation of ROS, especially under low K^+^ treatment.

### Treatment With the Ca^2+^ Channel Blockers and a Ca^2+^ Chelator Reduces Antioxidant Enzyme Activities and the Expression of Antioxidant Enzyme Genes in Tobacco Plant Roots Under Low-K^+^ Stress

**Figure 4:**
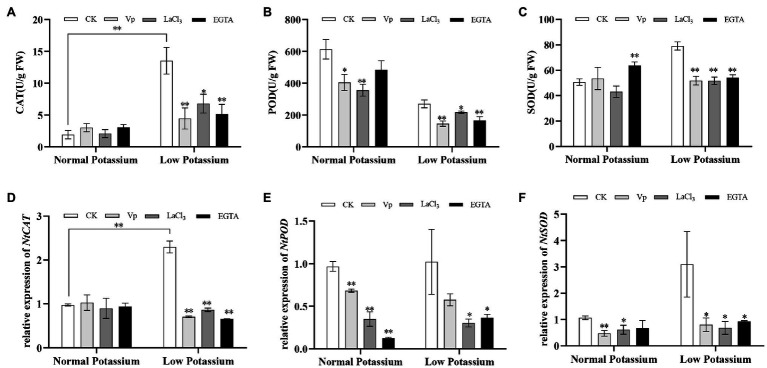
The activity levels of catalase (CAT; **A**), peroxidase (POD; **B**), and superoxide dismutase (SOD; **C**) in CK, blocker-treated, and chelator-treated tobacco plant roots under normal or low potassium levels. Transcriptional expression of *NtCAT* **(D)**, *NtPOD* **(E)**, and *NtSOD* **(F)** in CK, blocker-treated, and chelator-treated tobacco plant roots under normal and low potassium levels. * and ** indicate significant differences between CK and treated plants at *p* < 0.05 and *p* < 0.01, respectively, by Student’s *t*-test.

To clarify the mechanism by which ROS accumulation in tobacco roots is alleviated by Ca^2+^ signaling in the low K^+^ treatment, we measured the activities of several antioxidant enzymes in CK, blocker-treated, and chelator-treated plants under normal and low K^+^ levels. In the low K^+^ treatment, the activities of CAT, POD, and SOD were significantly decreased in blocker-treated and chelator-treated plants than in CK plants (Figures 4A–C). Interestingly, compared to their corresponding the normal potassium level, the CK plants showed a 7-fold increase in CAT upon low potassium treatment, whereas these blocker-treated plants showed an average increase of only 2-fold (Figure 4A). In the normal K^+^ level, CAT activity did not differ among treatments (Figure 4A). These results indicated that antioxidant enzymes, such as CAT, POD, and SOD, were activated by Ca^2+^ signaling under low-K^+^ stress. Next, we analyzed the transcript levels of these three antioxidant enzyme genes. The results were similar to those of antioxidant enzyme activities. In the low K^+^ treatment, the transcript levels of *NtCAT*, *NtPOD*, and *NtSOD* were significantly downregulated in blocker-treated and chelator-treated plants compared with CK plants (Figures 4D–F). In particular, the expression of *NtCAT* was significantly upregulated in CK plants in the low K^+^ treatment compared with the normal K^+^ treatment. These results, along with the changes in H_2_O_2_ and O^•^_2_^−^ levels, indicated that Ca^2+^ signaling reduced ROS accumulation in the low K^+^ treatment by enhancing antioxidant enzyme activities and the transcript levels of related genes.

### Treatment With the Ca^2+^ Channel Blockers and a Ca^2+^ Chelator Reduces the NADK and NADPase Activities in Tobacco Plant Roots Under Low-K^+^ Stress

**Figure 5:**
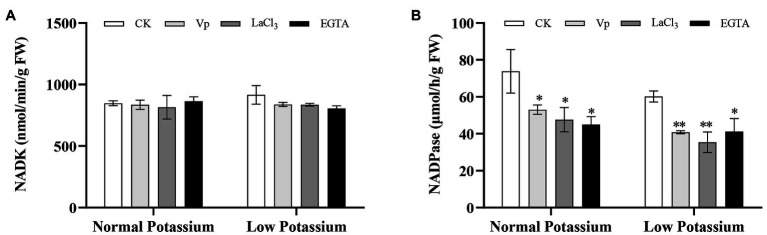
The activity levels of NADK **(A)** and NADPase **(B)** in tobacco plant roots in each treatment. * and ** indicate significant differences between CK and treated plants at *p* < 0.05 and *p* < 0.01, respectively, by Student’s *t*-test.

To characterize the effect of the different treatments on NAD signaling, NADK and NADPase activities were determined under normal and low K^+^ levels. NADK is a key enzyme for NADP production. NADPase can dephosphorylate NADP. In the low K^+^ treatment, plants treated with the Ca^2+^ channel blockers and a Ca^2+^ chelator had significantly lower NADK and NADPase activities in the roots than CK plants (Figure 5). NADK activity did not differ between treatments in the normal K^+^ treatment (Figure 5A). However, NADPase activity was significantly reduced in blocker-treated and chelator-treated plants compared with CK plants in the normal K^+^ treatment (Figure 5B). These results indicated that Ca^2+^ signaling can induce the key enzymes of NAD signaling and thereby affect plant K^+^ uptake under low-K^+^ stress.

### Treatment With the Ca^2+^ Channel Blockers and a Ca^2+^ Chelator Reduces the CaM Content in Tobacco Plant Roots Under Low-K^+^ Stress

**Figure 6:**
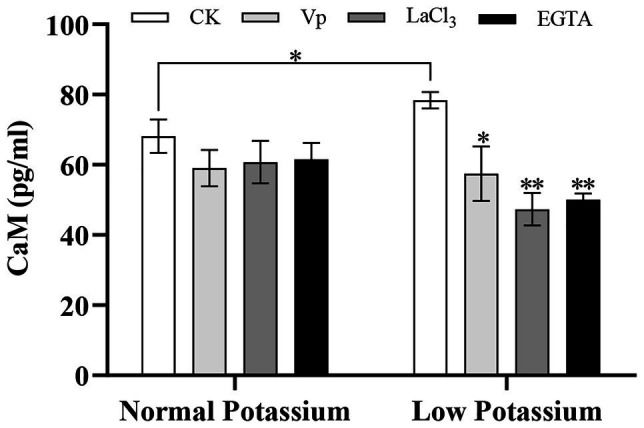
Effect of two Ca^2+^ channel blockers and a Ca^2+^ chelator on the calmodulin (CaM) content in tobacco roots under normal and low potassium treatments. * and ** indicate significant differences between CK and treated plants at *p* < 0.05 and *p* < 0.01, respectively, by Student’s *t*-test.

Many studies have shown that many Ca^2+^ signal sensors, such as CBL and CIPK, are involved in plant K^+^ uptake under low-K^+^ stress; however, no studies to date have examined whether CaM is involved in this process. The CaM content decreased in both the normal and low K^+^ treatments following the addition of blockers and a chelator (Figure 6). However, the magnitude of the decrease varied among treatments. For example, after low K^+^ treatment, the CaM content was considerably lower in blocker-treated and chelator-treated plants than in the CK plants, indicating that the CK plants accumulated a higher concentration of CaM relative to these blocker-treated plants after low-K^+^ treatment (Figure 6). There was no significant difference between treatments under the normal K^+^ level (Figure 6). Noticeably, the CaM content of the CK plants was significantly higher in the low K^+^ treatment than in the normal K^+^ treatment (Figure 6). This suggested that the regulation of CaM by Ca^2+^ signaling in plant roots under low-K^+^ stress may contribute to changes in the K^+^ concentration and that low-K^+^ stress promoted an increase in the CaM content.

### Treatment With the Ca^2+^ Channel Blockers and a Ca^2+^ Chelator Alters the Expression of K^+^ Channel‐ and Transporter-Related Genes in Tobacco Plant Roots Under Low-K^+^ Stress

**Figure 7:**
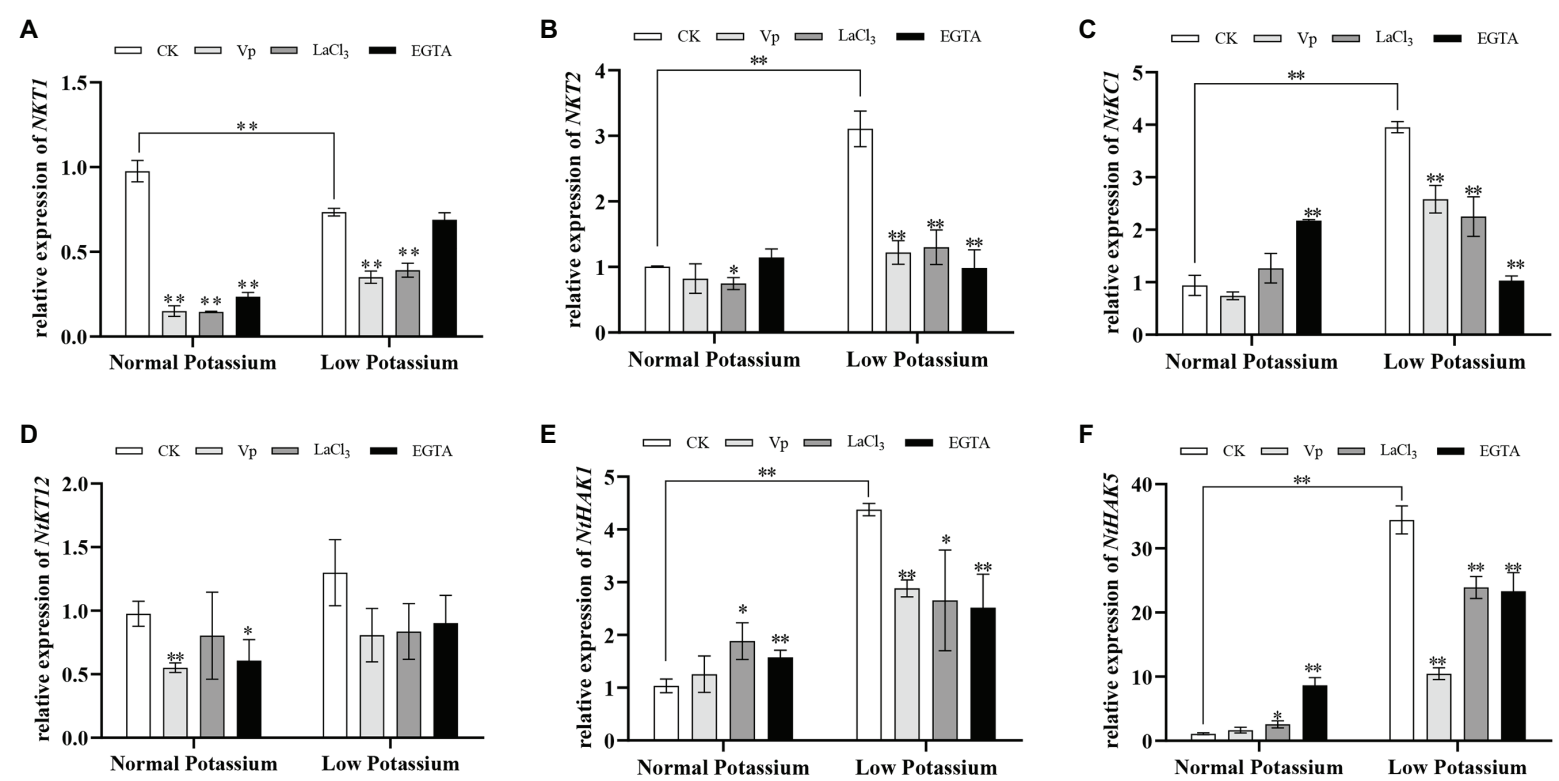
Expression of potassium channel genes (*NKT1*; **A**, *NKT2*; **B**, and *NtKC1*; **C**) and transporter genes (*NtKT12*; **D**, *NtHAK1*; **E**, and *NtHAK5*; **F**) in CK, blocker-treated, and chelator-treated tobacco plant roots under normal and low potassium levels, as detected by RT-qPCR. * and ** indicate significant differences between CK and treated plants at *p* < 0.05 and *p* < 0.01, respectively, by Student’s *t*-test.

To elucidate the molecular mechanisms by which the Ca^2+^ signaling pathway mediates K^+^ uptake in tobacco plants under low-K^+^ uptake stress, we determined the transcript levels of three K^+^ channel genes and three K^+^ transporter genes for K^+^ uptake in plants treated with Ca^2+^ channel blockers and a Ca^2+^ chelator under normal and low K^+^ levels. The expression of four of these genes was significantly upregulated in CK plants in the low K^+^ treatment compared with the normal treatment (Figures 7B,C,E,F). Noticeably, compared with the CK plants under the normal K^+^ level, the expression of the *NtHAK5* was increased by 3.4 times in the CK plants upon low K^+^ treatment. However, the transcriptional level of *NKT1* in CK plants was significantly lower in the low K^+^ treatment than in the normal K^+^ treatment (Figure 7A). In the low-K^+^ treatment, the expression of all six genes was significantly higher in CK plants than those in blocker-treated and chelator-treated plants (Figure 7). These data suggest that interference with Ca^2+^ transport under low K^+^-stress downregulates the expression of K^+^ channel and transporter genes. This, in turn, indicates that Ca^2+^ signaling mediates the transcriptional regulation of these genes under low-K^+^ stress, which in turn affects K^+^ uptake.

### Treatment With the Ca^2+^ Channel Blockers and a Ca^2+^ Chelator Alters the Expression of *NtCNGC3* and *NtCNGC10* in Tobacco Plant Roots Under Low-K^+^ Stress

**Figure 8:**
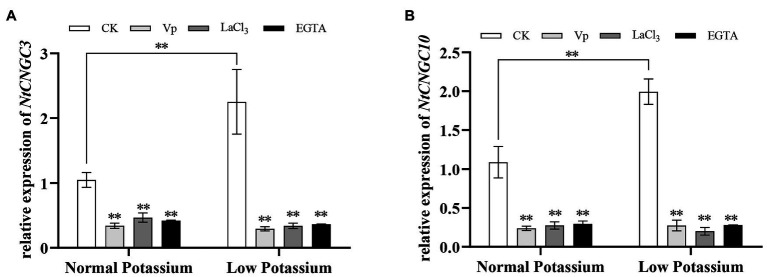
Expression of *NtCNGC3* (A) and *NtCNGC10* (B) in blocker-treated and chelator-treated tobacco plant roots and CK tobacco plant roots under normal and low potassium levels, as detected by RT-qPCR. **indicates significant differences between CK and treated plants at *p* < 0.01, by Student’s *t*-test.

Plant cyclic nucleotide-gated channels (CNGCs) are non-selective cation-conducting channels that facilitate the uptake of cations, including Ca^2+^ and K^+^. It has been reported that the CNGCs have been a possible pathway for K^+^ uptake (Li et al., 2005; Gobert et al., 2006; Caballero et al., 2012). Therefore, the transcript levels of *NtCNGC3* and *NtCNGC10* in the tobacco plant roots under various treatments were detected by real-time quantitative PCR (RT-qPCR). The expression of *NtCNGC3* and *NtCNGC10* was significantly higher in the roots of CK plants in the low K^+^ treatment than those in the normal K^+^ treatment (Figure 8). The expression levels of *NtCNGC3* and *NtCNGC10* were significantly reduced in plant roots treated with Ca^2+^ channel blockers and a Ca^2+^ chelator in both K^+^ treatments (Figure 8). These results indicate that *NtCNGC3* and *NtCNGC10* were involved in K^+^ uptake by tobacco roots under low-K^+^ stress and were regulated by Ca^2+^ signaling.

### Treatment With CaM Antagonists Reduces the K^+^ Concentration in Various Parts of Tobacco Plants Under Low-K^+^ Stress

**Figure 9:**
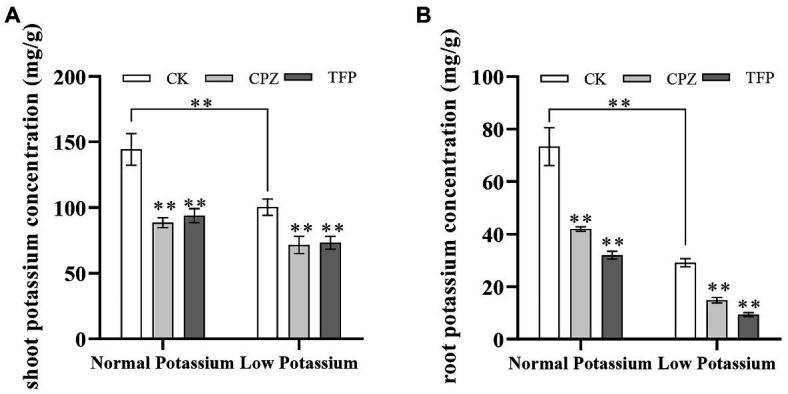
Effect of CaM antagonists on the potassium concentration in the shoots **(A)** and roots **(B)** of tobacco plants. **indicates significant differences between CK and treated plants at *p* < 0.01, by Student’s *t*-test.

The above results indicate that low-K^+^ stress induces an increase in CaM content and that this increase is positively regulated by Ca^2+^ signaling. We thus speculated that CaM might function as a Ca^2+^ signal sensor involved in the regulation of K^+^ uptake in plant roots under low-K^+^ stress. We examined whether CaM is involved in K^+^ uptake by plant roots under low-K^+^ stress by treating tobacco plants with CaM antagonists. We first determined the K^+^ concentration of CK and antagonist-treated plants. Our data showed that the K^+^ concentration was significantly lower in antagonist-treated plants than in CK plants in both K^+^ treatments (Figure 9). In the low K^+^ treatments, the K^+^ concentration of antagonist-treated plants was reduced by 39% on average in shoots and 95% on average in roots compared with CK plants (Figure 9). CaM antagonists significantly reduced K^+^ concentration in plant shoots and roots. These results further indicated that CaM is involved in K^+^ uptake by plant roots.

### Treatment With CaM Antagonists Increases ROS Accumulation in Tobacco Plant Roots Under Low-K^+^ Stress

**Figure 10:**
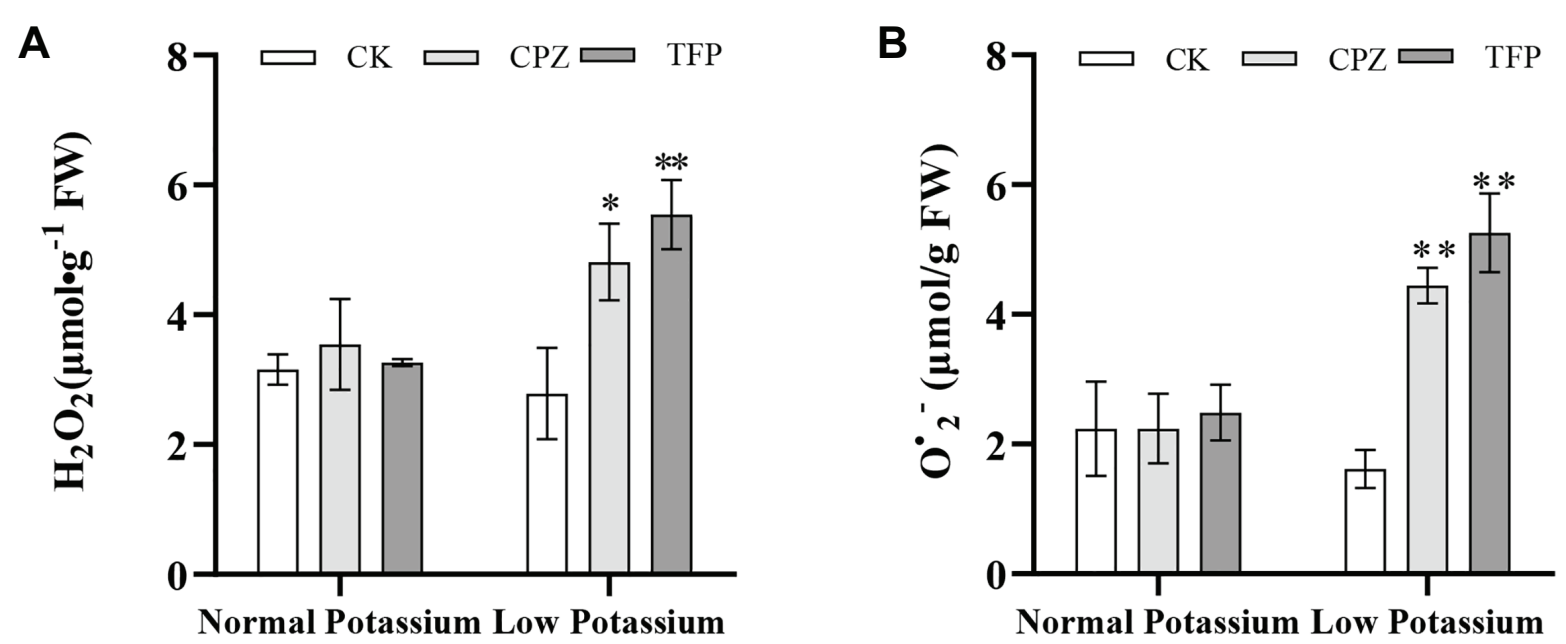
Effect of CaM antagonists on H_2_O_2_ **(A)** and O^•^_2_^−^ **(B)** in tobacco roots. ^*^ and ^**^ indicate significant differences between CK and treated plants at *p* < 0.05 and *p* < 0.01, respectively, by Student’s *t*-test.

To further confirm whether CaM mediates K^+^ uptake in tobacco plant roots by affecting ROS accumulation, ROS accumulation was measured under antagonist treatment. After antagonist treatment, the H_2_O_2_ and O^•^_2_^−^ levels were significant higher in antagonist-treated plants than in CK plants under low-K^+^ stress (Figure 10). However, there was no significant difference in the levels of H_2_O_2_ and O^•^_2_^−^ between CK and antagonist-treated plants in the normal K^+^ treatments (Figure 10). This result indicated that the CaM signaling pathway could reduce ROS accumulation under low-K^+^ stress.

### Treatment With CaM Antagonists Reduces Antioxidant Enzyme Activities in Tobacco Plant Roots Under Low-K^+^ Stress

**Figure 11:**
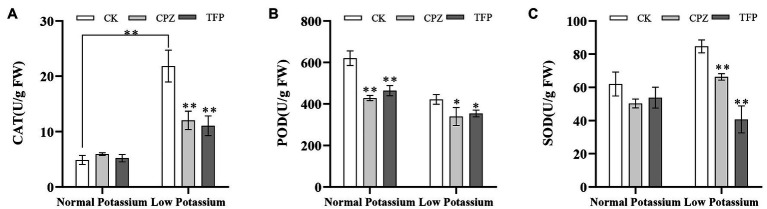
Changes in CAT **(A)**, POD **(B)**, and SOD **(C)** activity in CK and antagonist-treated tobacco plant roots under normal and low potassium levels. * and ** indicate significant differences between CK and treated plants at *p* < 0.05 and *p* < 0.01, respectively, by Student’s *t*-test.

We examined the activities of three antioxidant enzymes (CAT, POD, and SOD) in CK and antagonist-treated plant roots under normal and low K^+^ levels. In the low K^+^ treatment, antagonist-treated plants showed significant decreases in CAT, POD, and SOD activities compared with CK plants (Figure 11). POD activity was significantly lower in antagonist-treated plants than in CK plants in the normal K^+^ treatment (Figure 11B). Noticeably, CAT activity of the CK plants in low K^+^ treatment was significantly higher than the CK plants in normal K^+^ treatment (Figure 11A). The same pattern was observed among plants treated with Ca^2+^ channel blockers and a Ca^2+^ chelator. These results, coupled with changes in H_2_O_2_ and O^•^_2_^−^ levels following treatment with CaM antagonists, suggested that the CaM signaling pathway may reduce ROS accumulation by enhancing the activities of major antioxidant enzymes under low-K^+^ stress.

### Treatment With CaM Antagonists Reduces the NADK and NADPase Activities in Tobacco Plant Roots Under Low-K^+^ Stress

**Figure 12:**
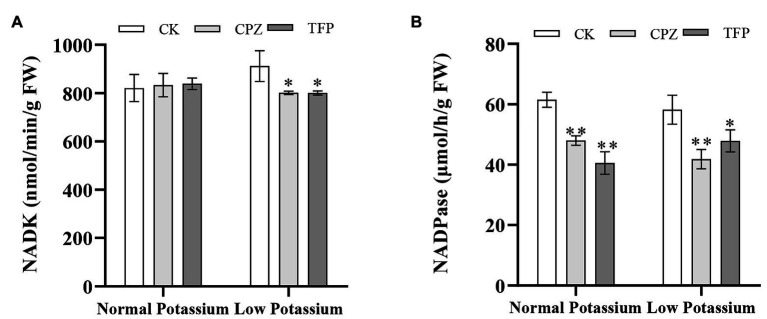
Changes in NADK (A) and NADPase (B) activity in CK and antagonist-treated tobacco plant roots under normal and low potassium levels. * and ** indicate significant differences between CK and treated plants at *p* < 0.05 and *p* < 0.01, respectively, by Student’s *t*-test.

The NADK and NADPase activities in CK and antagonist-treated plant roots under normal and low K^+^ levels were examined. After antagonist treatment, the NADK activity was significant higher in the CK plants than in antagonist-treated plants in the low K^+^ treatment (Figure 12A). Furthermore, the NADK activity in CK plants was higher in the low K^+^ treatment than in the normal K^+^ treatment (Figure 12A). NADPase activity was significantly inhibited by CaM antagonists at both K^+^ levels (Figure 12B). NADPase activity was significantly lower in antagonist-treated plants than that in CK plants at both normal and low K^+^ levels (Figure 12B). These results indicated that the application of CaM antagonists can reduce NADK and NADPase activities under low-K^+^ stress.

### Treatment With CaM Antagonists Alters the Expression of K^+^ Channel and Transporter Genes in Tobacco Plant Roots Under Low-K^+^ Stress

**Figure 13:**
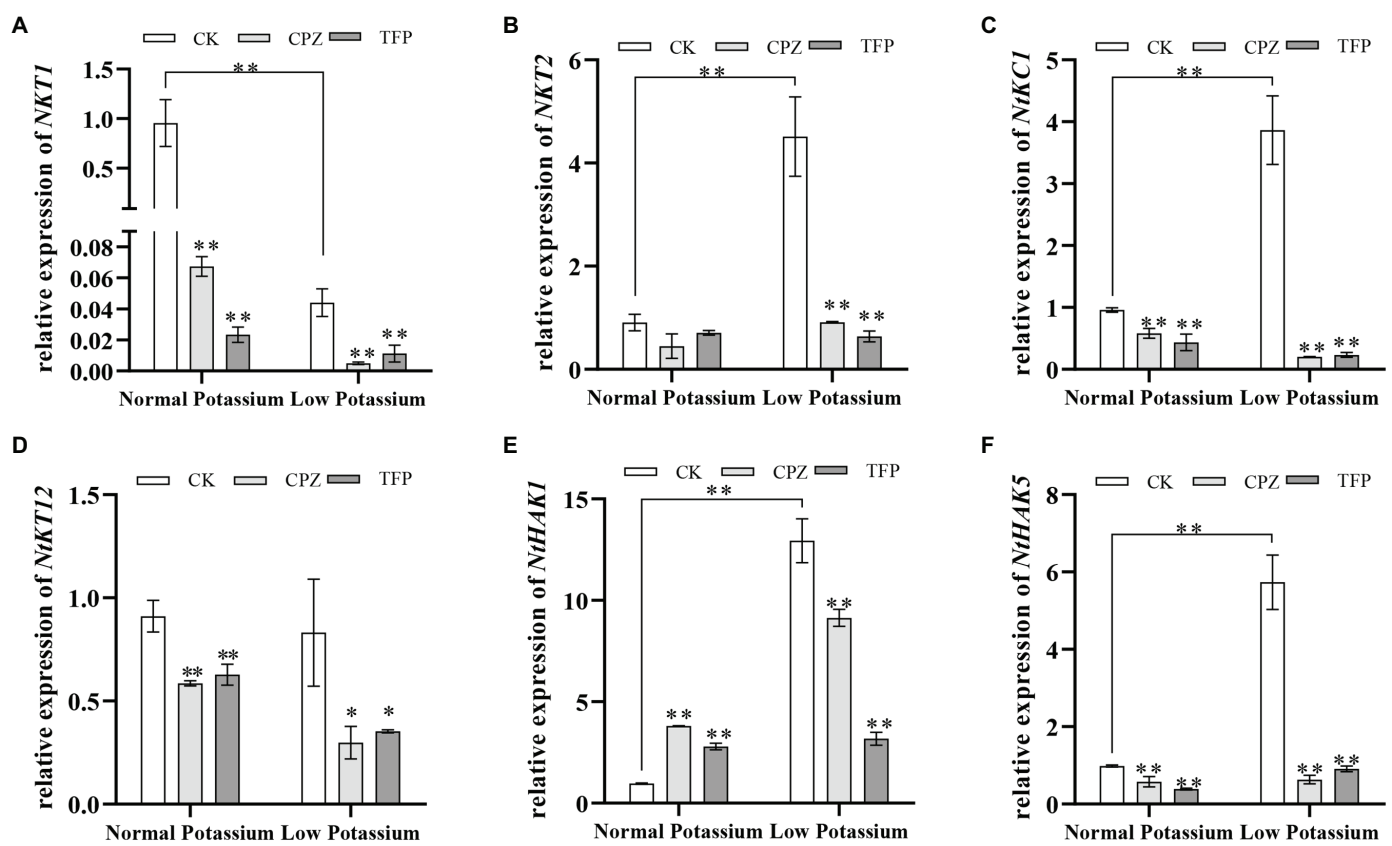
Expression of potassium channel genes (*NKT1*; **A**, *NKT2*; **B**, and *NtKC1*; **C**) and transporter genes (*NtKT12*; **D**, *NtHAK1*; **E**, and *NtHAK5*; **F**) in CK and antagonist-treated tobacco plant roots under normal and low potassium levels, as detected by RT-qPCR. ^*^ and ^**^ indicate significant differences between CK and treated plants at *p* < 0.05 and *p* < 0.01, respectively, by Student’s *t*-test.

To further explore the molecular mechanisms by which of CaM mediates K^+^ uptake, we characterized the transcriptional changes of three K^+^ channel genes (*NKT1*, *NKT2*, and *NtKC1*) and three transporter genes (*NtKT12*, *NtHAK1*, and *NtHAK5*) in both CK and antagonist-treated plants by RT-qPCR under normal and low potassium levels. The expression levels of *NKT2*, *NtKC1*, *NtHAK1*, and *NtHAK5* were much higher in CK plants in the low K^+^ treatment than in the normal K^+^ treatment (Figures 13B,C,E,F). Notably, in the low K^+^ treatment, the transcript abundance of the six genes was significantly decreased in the antagonist-treated plants compared with CK plants (Figure 13). These data indicate that the application of CaM antagonists can inhibit the expression of K^+^ channel genes and transporter genes under low K^+^-stress.

### Treatment With CaM Antagonists Alters the Expression of *NtCNGC3* and *NtCNGC10* in Tobacco Plant Roots Under Low-K^+^ Stress

**Figure 14:**
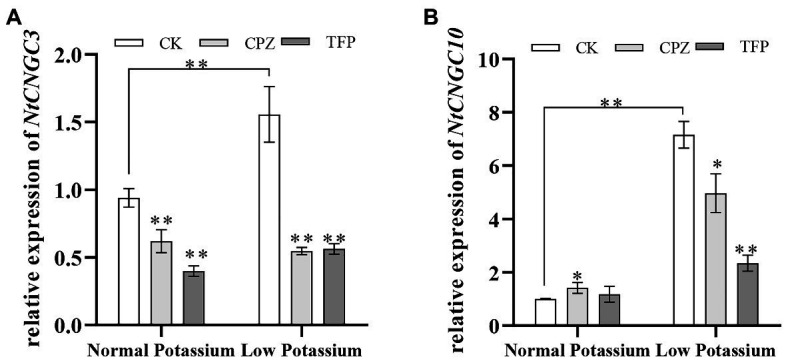
Expression of *NtCNGC3* (A) and *NtCNGC10* (B) in antagonist-treated tobacco plant roots and CK tobacco plant roots under normal and low potassium levels, as detected by RT-qPCR. * and ** indicate significant differences between CK and treated plants at *p* < 0.05 and *p* < 0.01, respectively, by Student’s *t*-test.

Previous studies have shown that Ca^2+^ signaling can affect the expression of *NtCNGC3* and *NtCNGC10* under low-K^+^ stress. We thus measured the transcriptional changes in *NtCNGC3* and *NtCNGC10* following the application of CaM antagonists. The expression of *NtCNGC3* and *NtCNGC10* was significantly increased in CK plants in the low K^+^ treatment compared with the normal K^+^ treatment (Figure 14). The antagonist-treated plants had significantly lower expression of *NtCNGC3* and *NtCNGC10* compared with CK plants in the low K^+^ treatment (Figure 14). This finding suggested that CaM might affect plant K^+^ uptake under low K^+^-stress by affecting the expression of *NtCNGC3* and *NtCNGC10*.

## Discussion

Potassium is an important macroelement affecting plant growth. Identifying the signaling cascades involved in plant K^+^ uptake under K^+^ deficiency is thus critically important for understanding their mechanisms of action. CaM is an important Ca^2+^ sensor protein in plants that plays a role in responses to different biotic and abiotic stresses. No studies to date have assessed whether the Ca^2+^-CaM signaling pathway is involved in K^+^ uptake by plant roots under low-K^+^ stress. In this study, we demonstrated that the Ca^2+^-CaM signaling pathway is involved in the response to K^+^ deficiency and K^+^ uptake in roots by possibly modulating ROS homeostasis; the activity of antioxidant enzymes, NAD kinase, and NADP phosphatase; and the expression of *NtCNGC3*, *NtCNGC10*, and K^+^ channel genes and transporter genes.

The concentration and distribution of cytosolic free Ca^2+^ are key to Ca^2+^ signaling. The intracellular free Ca^2+^ concentration has been reported to increase in plants in response to different biotic and abiotic stresses (Bose et al., 2011). K^+^ deficiency triggers spatially and temporally defined elevation of Ca^2+^ concentration in roots, which represents a key response at low-K^+^ condition (Behera et al., 2017). K^+^ deficiency induces increased Ca^2+^ concentration in guard cells (Allen et al., 2001) and pollen tubes (Zhao et al., 2013) of *Arabidopsis*. Changes in the free Ca^2+^ concentration in the cytoplasm are recognized, decoded, and further transmitted by various Ca^2+^ sensors. Intracellular Ca^2+^ is released from extracellular pools or intracellular stores into the cytosol by various Ca^2+^ channels and transporters on cell organelles and/or membranes and is pumped back to organelles and apoplasts by Ca^2+^-ATPase (Ca^2+^ pumps) and Ca^2+^/H^+^ antiports (Yang and Poovaiah, 2003). Following K^+^ deficiency, the hyperpolarization of PM can activate Ca^2+^ channel located within root epidermis and root hairs zone (Véry and Davies, 2000; Demidchik et al., 2002). Calcium channel blockers, such as VP and LaCl_3_, can block the entry of extracellular Ca^2+^ into cells and alter the intracellular Ca^2+^ concentration. EGTA is a Ca^2+^ chelator that can chelate extracellular Ca^2+^, thus reducing the concentration of free extracellular Ca^2+^ and Ca^2+^ in the cytoplasm. In this study, we showed that inhibition of the intracellular Ca^2+^ concentration significantly inhibited the K^+^ concentration the in shoots and roots of tobacco. These findings suggest that intracellular Ca^2+^ plays an important role in the low-K^+^ response in tobacco. Intracellular Ca^2+^ signaling under low-K^+^ stress facilitates the detection of K^+^ deficiency by plant roots and promotes the uptake of K^+^.

Reactive oxygen species play an important role in several signal transduction pathways. ROS also accumulate in roots in the absence of nitrogen, phosphorus, and K^+^ (Shin et al., 2005). However, the excessive accumulation of ROS can lead to oxidative damage within cells. Thus, strict control of the concentration of ROS, while permitting ROS (especially H_2_O_2_) to perform useful signaling functions under stress conditions is critically important (Hernandez et al., 2010). The roots of blocker-treated and chelator-treated tobacco plants accumulated more H_2_O_2_ and O^•^_2_^−^ than CK roots in the normal and low K^+^ treatments, suggesting that intracellular Ca^2+^ might contribute to K^+^ uptake by regulating ROS accumulation in roots. Furthermore, the activity of CAT, POD, and SOD in blocker-treated and chelator-treated roots in the low K^+^ level was decreased. CAT, POD, and SOD are three scavenging enzymes necessary for the detoxification of ROS. The expression levels of the antioxidant-related genes *NtCAT*, *NtPOD*, and *NtSOD* were significantly downregulated in plants treated with channel blockers and a chelator under low-K^+^ stress. These data suggest that intracellular Ca^2+^ might regulate the accumulation of ROS by activating these antioxidant enzymes, thereby improving the K^+^ uptake capacity of roots under low-K^+^ stress. Consistent with our results, EGTA and LaCl_3_ have been shown to significantly suppress the activity of antioxidant enzymes (Niu et al., 2017). Several studies have suggested that both long-term and short-term K^+^ starvation can cause ROS accumulation in plant roots and oxidative damage (Banuelos et al., 2002; Shin and Schachtman, 2004). ROS stress induces K^+^ leakage in plant tissues (Quartacci et al., 2001; Demidchik et al., 2003), and higher antioxidant enzyme activities are required to tolerate K^+^ deficiency (Tewari et al., 2007; Hafsi et al., 2011). Intracellular Ca^2+^ signaling may play an important role in this process by mitigating oxidative damage in plant cells and mediating the signaling function of ROS, which promotes K^+^ uptake by plant roots under low-K^+^ stress.

To understand the roles that two Ca^2+^ channel blockers and a Ca^2+^ chelator playing in reducing the K^+^ concentration in tobacco, the expression of K^+^ channel and K^+^ transporter genes was studied. The expression of all of the three K^+^ channel genes and three K^+^ transporter genes were repressed in the roots of the treated tobacco plants relative to CK plants in the low K^+^ level. *NKT1*, *NKT2*, and *NtKC1* encode inwardly rectifying K^+^ channel proteins (Dai et al., 2009). Studies on the relationship between the Ca^2+^ signaling pathway and K^+^ channels have shown that the interaction of Ca^2+^ sensors (CBL1 and CBL9) with target kinase CIPK23 under low-K^+^ stress enhances K^+^ uptake by activating the *AKT1* channel in a Ca^2+^-dependent manner (Li et al., 2006). Intracellular Ca^2+^ signaling can also differentially affect the activity of *AKT1*, *AKT2*, and *AtKC1* to mediate K^+^ uptake by *Arabidopsis* under low-K^+^ stress (Cheong et al., 2007; Held et al., 2011; Wang et al., 2016). KT12, HAK1, and HAK5 are KT/KUP/HAK-type transporters that are thought to play a role in high-affinity and/or low-affinity K^+^ transport (Very and Sentenac, 2003). Studies of *Arabidopsis* have shown that HAK5 is activated *in vivo* by *AtCBL1*/*AtCIPK23* and is the main transporter that regulates K^+^ uptake when the external K^+^ concentration is low (<10 μM; Held et al., 2011). Under low-K^+^ stress, the application of the Ca^2+^ channel blockers and a Ca^2+^ chelator inhibited intracellular Ca^2+^ signaling and the decoding and transmission of Ca^2+^ sensors, which affected the expression of K^+^ channel and transporter genes and led to changes in K^+^ uptake and the K^+^ concentration in plants. Therefore, the Ca^2+^ signaling pathway plays an important role in K^+^ uptake by plant roots under low-K^+^ stress by upregulating the expression of several K^+^ channel genes and transporter genes. We also found that the expression of some K^+^ channel genes (*NKT2* and *NtKC1*) was upregulated under low-K^+^ stress, which indicated that not only K^+^ transporters but also K^+^ channels were involved in K^+^ uptake under low-K^+^ stress.

Cyclic nucleotide-gated channels are a large group of nonspecific cation channels in plants (Demidchik et al., 2002). CNGCs, such as CNGC3 and CNGC10, have been shown to have K^+^ inward-rectifying channel activity and are involved in root K^+^ uptake (Leng et al., 2002; Li et al., 2005; Gobert et al., 2006; Ma et al., 2006). Our results indicate that the transcripts of *NtCNGC3* and *NtCNGC10* were significantly upregulated during low-K^+^ stress, and the Ca^2+^ channel blockers and a Ca^2+^ chelator significantly inhibited the expressions of those genes. Therefore, we speculate that *NtCNGC3* and *NtCNGC10* may be involved in K^+^ uptake by tobacco roots under low-K^+^ stress in a Ca^2+^-dependent manner. Consistent with this speculation, K^+^ uptake by *akt1* mutants was shown to be complemented by CNGC3 and CNGC10 (Caballero et al., 2012). Similarly, K^+^ uptake was significantly inhibited in cngc3 plants, and overexpression of *AtCNGC10* genes partially complemented the mutant for K^+^ uptake; however, the antisense of *AtCNGC10* resulted in a 40% reduction in the K^+^ concentration (Kaplan et al., 2007). Overall, this indicates that CNGC3 and CNGC10 (and other CNGCs) may be involved in K^+^ uptake in plant roots under low-K^+^ stress, which is mediated by Ca^2+^ signaling.

Noticeably, in our study, the CaM content was significantly increased in the low K^+^ treatment; however, the application of Ca^2+^ channel blockers and a Ca^2+^ chelator decreased the CaM content in the low K^+^ treatment. CaM is a major Ca^2+^ sensor that plays a key role in the decoding and transmission of Ca^2+^ sensors (Zeng et al., 2015). However, no studies to date have examined whether CaM is involved in K^+^ uptake in plants. We treated tobacco plants with CaM antagonists. Interestingly, we found that K^+^ concentration in various parts of tobacco plants was significantly reduced after the application of CaM antagonists. These results strongly suggest that the Ca^2+^-CaM signaling pathway is involved in K^+^ uptake in tobacco plants. Furthermore, ROS accumulation was higher and antioxidant enzyme activity lower in CaM-antagonists treated roots than in CK roots in low K^+^ treatment. Therefore, CaM can reduce ROS accumulation by increasing the activity of major antioxidant enzymes in the roots of tobacco plants. Consistent with our findings, Larkindale and Knight (2002) showed that CaM antagonists can aggravate oxidative damage in *Arabidopsis* seedlings under heat stress (Larkindale and Knight, 2002). The Ca^2+^-CaM signaling pathway also plays an important role in balancing ROS. For example, studies of heat-stressed maize seedlings have shown that both Ca^2+^ influx and intracellular CaM can regulate seedling ROS homeostasis and the antioxidant system, including CAT, SOD, and APX (Gong et al., 1997). In our study, Ca^2+^ and CaM could both affect the activity of NADK and NADPase, especially under low-K^+^ stress. Given that they are target enzymes of the Ca^2+^-CaM complex, we speculate that NADK and NADPase are involved in regulating ROS homeostasis in plant roots under low-K^+^ stress. The NADP(H)/NAD(H) ratio functions in ROS generation and scavenging and biological processes in cells, such as signal transduction and energy metabolism, and is regulated by the key regulators NADK and NADPase (Richter, 1987; Hashida et al., 2018; Li et al., 2018). NADPH is a product of NADK and a substrate of NADPase that plays a dual role in ROS homeostasis and is mediated by the Ca^2+^-CaM complex (Roberts and Harmon, 1992; Mittler, 2002; Bedard et al., 2007). The transcript abundance of *NtCNGC3*, *NtCNGC10*, and K^+^ channel genes and transporter genes treated with CaM antagonists were similar to those treated with two Ca^2+^ channel blockers and a Ca^2+^ chelator. Under low K^+^-stress, the application of CaM antagonists significantly inhibited the expression of these genes involved in K^+^ uptake in plants. Thus, CaM may play an important role in perceiving, decoding, and further transmitting Ca^2+^ signals to downstream target proteins during the low-K^+^ responses.

**Figure 15:**
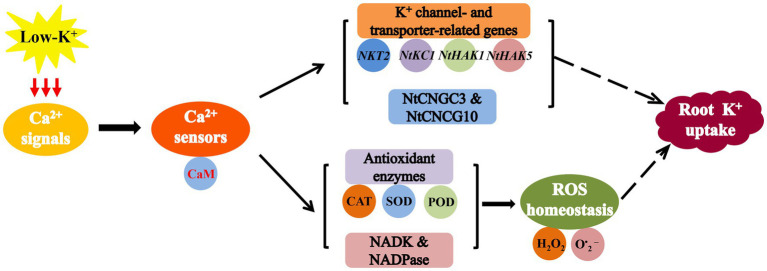
A model for the role of the Ca^2+^-CaM signaling pathway in regulating potassium uptake in plant roots under low-K^+^ stress. Ca^2+^-CaM signaling pathway upregulates expression of several K^+^ channel genes and K^+^ transporter genes (*NKT2*, *NtKC1*, *NtHAK1*, and *NtHAK5*), *NtCNGC3*, and *NtCNGC10*. It also mediates reactive oxygen species (ROS) homeostasis by increasing the activity of CAT, SOD, POD, NADK, and NADPase.

In conclusion, our data demonstrated that the Ca^2+^-CaM signaling pathway in plant roots might mediate ROS homeostasis and promote K^+^ uptake by plant roots by increasing the activity of several major antioxidant enzymes, NADK, and NADPase under low-K^+^ stress (Figure 15). Meanwhile, coupled with the results of the gene expression, suggested that the intracellular Ca^2+^-CaM signaling pathway positively regulates low-K^+^ stress root K^+^ uptake at least partly through upregulation of the expression of *NtCNGC3*, *NtCNGC10*, K^+^ channel genes, and K^+^ transporter genes in plant roots (Figure 15). Generally, the results of this study provide new insights into the role of Ca^2+^ signals and CaM in regulateing K^+^ uptake in plant roots under low-K^+^ stress.

## Data Availability Statement

The raw data supporting the conclusions of this article will be made available by the authors, without undue reservation.

## Author Contributions

YW and XD: conceptualization and writing. YW, GX, PC, ZD, TZ, and HZ: investigation. YW and GX: data analysis. XD: project administration and supervision. All authors contributed to the article and approved the submitted version.

### Conflict of Interest

The authors declare that the research was conducted in the absence of any commercial or financial relationships that could be construed as a potential conflict of interest.

## References

[ref1] Allen G. J. Chu S. P. Harrington C. L. Schumacher K. Hoffmann T. Tang Y. Y.. (2001). A defined range of guard cell calcium oscillation parameters encodes stomatal movements. Nature 411, 1053–1057. 10.1038/35082575, PMID: 11429606

[ref2] Ashley M. K. Grant M. Grabov A. (2006). Plant responses to potassium deficiencies: a role for potassium transport proteins. J. Exp. Bot. 57, 425–436. 10.1093/jxb/erj034, PMID: 16364949

[ref3] Banuelos M. A. Garciadeblas B. Cubero B. Rodrıguez-Navarro A. (2002). Inventory and functional characterization of the HAK potassium transporters of rice. Plant Physiol. 130, 784–795. 10.1104/pp.007781, PMID: 12376644 PMC166606

[ref4] Bedard K. Lardy B. Krause K. H. (2007). NOX family NADPH oxidases: not just in mammals. Biochimie 89, 1107–1112. 10.1016/j.biochi.2007.01.012, PMID: 17400358

[ref5] Behera S. Long Y. Schmitz-Thom I. Wang X. P. Zhang C. Li H.. (2017). Two spatially and temporally distinct Ca^2+^ signals convey *Arabidopsis thaliana* responses to K^+^ deficiency. New Phytol. 213, 739–750. 10.1111/nph.14145, PMID: 27579668

[ref6] Bose J. Pottosin I. Shabala S. S. S. Palmgren M. G. Shabala S. (2011). Calcium efflux systems in stress signaling and adaptation in plants. Front. Plant Sci. 2:85. 10.3389/fpls.2011.00085, PMID: 22639615 PMC3355617

[ref7] Bradford M. M. (1976). A rapid and sensitive method for the quantitation of microgram quantities of protein utilizing the principle of protein-dye binding. Anal. Biochem. 72, 248–254. 10.1016/0003-2697(76)90527-3, PMID: 942051

[ref8] Caballero F. Botella M. A. Rubio L. Fernández J. A. Martínez V. Rubio F. (2012). A Ca^2+^-sensitive system mediates low-affinity K^+^ uptake in the absence of AKT1 in *Arabidopsis* plants. Plant Cell Physiol. 53, 2047–2059. 10.1093/pcp/pcs140, PMID: 23054389

[ref9] Chai M. F. Chen Q. J. An R. Chen Y. M. Chen J. Wang X. C. (2005). NADK2, an *Arabidopsis* chloroplastic NAD kinase, plays a vital role in both chlorophyll synthesis and chloroplast protection. Plant Mol. Biol. 59, 553–564. 10.1007/s11103-005-6802-y, PMID: 16244906

[ref10] Cheong Y. H. Pandey G. K. Grant J. J. Batistic O. Li L. Kim B. G.. (2007). Two calcineurin B-like calcium sensors, interacting with protein kinase CIPK23, regulate leaf transpiration and root potassium uptake in *Arabidopsis*. Plant J. 52, 223–239. 10.1111/j.1365-313X.2007.03236.x, PMID: 17922773

[ref11] Dai X. Y. Su Y. R. Wei W. X. Wu J. S. Fan Y. K. (2009). Effects of top excision on the potassium accumulation and expression of potassium channel genes in tobacco. J. Exp. Bot. 60, 279–289. 10.1093/jxb/ern285, PMID: 19112172

[ref12] Delumeau O. Paven M. C. M. L. Montrichard F. Laval-Martin D. L. (2000). Effects of short-term NaCl stress on calmodulin transcript levels and calmodulin-dependent NAD kinase activity in two species of tomato. Plant Cell Environ. 23, 329–336. 10.1046/j.1365-3040.2000.00545.x

[ref13] Demidchik V. Davenport R. J. Tester M. (2002). Nonselective cation channels in plants. Annu. Rev. Plant Biol. 53, 67–107. 10.1146/annurev.arplant.53.091901.161540, PMID: 12221989

[ref14] Demidchik V. Maathuis F. J. (2007). Physiological roles of nonselective cation channels in plants: from salt stress to signalling and development. New Phytol. 175, 387–404. 10.1111/j.1469-8137.2007.02128.x, PMID: 17635215

[ref15] Demidchik V. Shabala S. N. Coutts K. B. Tester M. A. Davies J. M. (2003). Free oxygen radicals regulate plasma membrane Ca^2+^-and K^+^-permeable channels in plant root cells. J. Cell Sci. 116, 81–88. 10.1242/jcs.00201, PMID: 12456718

[ref16] Demidchik V. Shabala S. N. Davies J. M. (2007). Spatial variation in H_2_O_2_ response of *Arabidopsis thaliana* root epidermal Ca^2+^ flux and plasma membrane Ca^2+^ channels. Plant J. 49, 377–386. 10.1111/j.1365-313X.2006.02971.x, PMID: 17181775

[ref17] Gierth M. Mäser P. Schroeder J. I. (2005). The potassium transporter *AtHAK5* functions in K^+^ deprivation-induced high-affinity K^+^ uptake and *AKT1* K^+^ channel contribution to K^+^ uptake kinetics in *Arabidopsis* roots. Plant Physiol. 137, 1105–1114. 10.1104/pp.104.057216, PMID: 15734909 PMC1065410

[ref18] Gobert A. Park G. Amtmann A. Sanders D. Maathuis F. J. (2006). *Arabidopsis thaliana* cyclic nucleotide gated channel 3 forms a non-selective ion transporter involved in germination and cation transport. J. Exp. Bot. 57, 791–800. 10.1093/jxb/erj064, PMID: 16449377

[ref19] Gong M. Chen S. N. Song Y. Q. Li Z. G. (1997). Effect of calcium and calmodulin on intrinsic heat tolerance in relation to antioxidant systems in maize seedlings. Funct. Plant Biol. 24, 371–379. 10.1071/PP96118

[ref20] Gong M. Li Z. G. (1995). Calmodulin-binding proteins from *Zea mays* germs. Phytochemistry 40, 1335–1339. 10.1016/0031-9422(95)00381-G

[ref21] Hafsi C. Romero-Puertas M. C. Luis A. Abdelly C. Sandalio L. M. (2011). Antioxidative response of *Hordeum maritimum* L. to potassium deficiency. Acta Physiol. Plant. 33, 193–202. 10.1007/s11738-010-0537-3

[ref22] Hasanuzzaman M. Bhuyan M. H. M. Nahar K. Hossain M. Mahmud J. A. Hossen M.. (2018). Potassium: a vital regulator of plant responses and tolerance to abiotic stresses. Agronomy 8:31. 10.3390/agronomy8030031

[ref23] Hashida S. N. Miyagi A. Nishiyama M. Yoshida K. Hisabori T. Kawai-Yamada M. (2018). Ferredoxin/thioredoxin system plays an important role in the chloroplastic NADP status of *Arabidopsis*. Plant J. 95, 947–960. 10.1111/tpj.14000, PMID: 29920827

[ref24] Hashida S. N. Takahashi H. Uchimiya H. (2009). The role of NAD biosynthesis in plant development and stress responses. Ann. Bot. 103, 819–824. 10.1093/aob/mcp019, PMID: 19201765 PMC2707885

[ref25] Held K. Pascaud F. Eckert C. Gajdanowicz P. Hashimoto K. Corratgé-Faillie C.. (2011). Calcium-dependent modulation and plasma membrane targeting of the AKT2 potassium channel by the CBL4/CIPK6 calcium sensor/protein kinase complex. Cell Res. 21, 1116–1130. 10.1038/cr.2011.50, PMID: 21445098 PMC3193494

[ref26] Hernandez M. Fernandez-Garcia N. Diaz-Vivancos P. Olmos E. (2010). A different role for hydrogen peroxide and the antioxidative system under short and long salt stress in *Brassica oleracea* roots. J. Exp. Bot. 61, 521–535. 10.1093/jxb/erp321, PMID: 19906795 PMC2803216

[ref27] Hernandez M. Fernandez-Garcia N. Garcia-Garma J. Rubio-Asensio J. S. Rubio F. Olmos E. (2012). Potassium starvation induces oxidative stress in *Solanum lycopersicum* L. roots. J. Plant Physiol. 169, 1366–1374. 10.1016/j.jplph.2012.05.015, PMID: 22771251

[ref28] Jeanguenin L. Alcon C. Duby G. Boeglin M. Chérel I. Gaillard I.. (2011). AtKC1 is a general modulator of *Arabidopsis* inward Shaker channel activity. Plant J. 67, 570–582. 10.1111/j.1365-313X.2011.04617.x, PMID: 21518051

[ref29] Kaplan B. Sherman T. Fromm H. (2007). Cyclic nucleotide-gated channels in plants. FEBS Lett. 581, 2237–2246. 10.1016/j.febslet.2007.02.017, PMID: 17321525

[ref30] Kim M. C. Chung W. S. Yun D. J. Cho M. J. (2009). Calcium and calmodulin-mediated regulation of gene expression in plants. Mol. Plant 2, 13–21. 10.1093/mp/ssn091, PMID: 19529824 PMC2639735

[ref31] Kim M. J. Ciani S. Schachtman D. P. (2010). A peroxidase contributes to ROS production during *Arabidopsis* root response to potassium deficiency. Mol. Plant 3, 420–427. 10.1093/mp/ssp121, PMID: 20139158

[ref32] Kirkman H. N. Gaetani G. F. (1984). Catalase: a tetrameric enzyme with four tightly bound molecules of NADPH. Proc. Natl. Acad. Sci. U. S. A. 81, 4343–4347. 10.1073/pnas.81.14.4343, PMID: 6589599 PMC345585

[ref33] Kurosaki F. (1997). Role of inward K^+^ channel located at carrot plasma membrane in signal cross-talking of cAMP with Ca^2+^ cascade. FEBS Lett. 408, 115–119. 10.1016/S0014-5793(97)00403-1, PMID: 9180280

[ref34] Kurosaki F. Kaburaki H. Nishi A. (1994). Involvement of plasma membrane-located calmodulin in the response decay of cyclic nucleotide-gated cation channel of cultured carrot cells. FEBS Lett. 340, 193–196. 10.1016/0014-5793(94)80136-3, PMID: 8131844

[ref35] Lan W. Z. Lee S. C. Che Y. F. Jiang Y. Q. Luan S. (2011). Mechanistic analysis of AKT1 regulation by the CBL-CIPK-PP2CA interactions. Mol. Plant 4, 527–536. 10.1093/mp/ssr031, PMID: 21596690

[ref36] Larkindale J. Knight M. R. (2002). Protection against heat stress-induced oxidative damage in *Arabidopsis* involves calcium, abscisic acid, ethylene, and salicylic acid. Plant Physiol. 128, 682–695. 10.1104/pp.010320, PMID: 11842171 PMC148929

[ref37] Leigh R. A. Wyn Jones R. G. (1984). A hypothesis relating critical potassium concentrations for growth to the distribution and functions of this ion in the plant cell. New Phytol. 97, 1–13. 10.1111/j.1469-8137.1984.tb04103.x

[ref38] Leng Q. Mercier R. W. Hua B. G. Fromm H. Berkowitz G. A. (2002). Electrophysiological analysis of cloned cyclic nucleotide-gated ion channels. Plant Physiol. 128, 400–410. 10.1104/pp.010832, PMID: 11842144 PMC148903

[ref39] Li X. Borsics T. Harrington H. M. Christopher D. A. (2005). *Arabidopsis* AtCNGC10 rescues potassium channel mutants of *E. coli*, yeast and *Arabidopsis* and is regulated by calcium/calmodulin and cyclic GMP in *E. coli*. Funct. Plant Biol. 32, 643–653. 10.1071/FP04233, PMID: 32689163

[ref40] Li L. Kim B. G. Cheong Y. H. Pandey G. K. Luan S. (2006). A Ca^2+^ signaling pathway regulates a K^+^ channel for low-K response in *Arabidopsis*. Proc. Natl. Acad. Sci. U. S. A. 103, 12625–12630. 10.1073/pnas.0605129103, PMID: 16895985 PMC1567929

[ref41] Li B. B. Wang X. Tai L. Ma T. T. Shalmani A. Liu W. T.. (2018). NAD kinases: metabolic targets controlling redox co-enzymes and reducing power partitioning in plant stress and development. Front. Plant Sci. 9:379. 10.3389/fpls.2018.01959, PMID: 29662499 PMC5890153

[ref42] Li J. Wu W. H. Wang Y. (2017). Potassium channel AKT1 is involved in the auxin-mediated root growth inhibition in *Arabidopsis* response to low K^+^ stress. J. Integr. Plant Biol. 59, 895–909. 10.1111/jipb.12575, PMID: 28782920

[ref43] Liu Z. Wang P. Zhang T. Li Y. Wang Y. Gao C. (2018). Comprehensive analysis of BpHSP genes and their expression under heat stresses in *Betula platyphylla*. Environ. Exp. Bot. 152, 167–176. 10.1016/j.envexpbot.2018.04.011

[ref44] Luan S. (2009). The CBL-CIPK network in plant calcium signaling. Trends Plant Sci. 14, 37–42. 10.1016/j.tplants.2008.10.005, PMID: 19054707

[ref45] Ma W. Ali R. Berkowitz G. A. (2006). Characterization of plant phenotypes associated with loss-of-function of AtCNGC1, a plant cyclic nucleotide gated cation channel. Plant Physiol. Biochem. 44, 494–505. 10.1016/j.plaphy.2006.08.007, PMID: 17027276

[ref46] Ma X. H. Xu J. Y. Han D. Huang W. X. Dang B. J. Jia W.. (2020). Combination of β-aminobutyric acid and Ca^2+^ alleviates chilling stress in tobacco (*Nicotiana tabacum* L.). Front. Plant Sci. 11:556. 10.3389/fpls.2020.00556, PMID: 32477386 PMC7237732

[ref47] Mittler R. (2002). Oxidative stress, antioxidants and stress tolerance. Trends Plant Sci. 7, 405–410. 10.1016/S1360-1385(02)02312-9, PMID: 12234732

[ref48] Niu L. Yu J. Liao W. Yu J. Zhang M. Dawuda M. M. (2017). Calcium and calmodulin are involved in nitric oxide-induced adventitious rooting of cucumber under simulated osmotic stress. Front. Plant Sci. 8:1684. 10.3389/fpls.2017.01684, PMID: 29021804 PMC5623940

[ref49] Okada T. Nakayama H. Shinmyo A. Yoshida K. (2008). Expression of OsHAK genes encoding potassium ion transporters in rice. Plant Biotechnol. 25, 241–245. 10.5511/plantbiotechnology.25.241

[ref50] Quartacci M. F. Cosi E. Navari-Izzo F. (2001). Lipids and NADPH-dependent superoxide production in plasma membrane vesicles from roots of wheat grown under copper deficiency or excess. J. Exp. Bot. 52, 77–84. 10.1093/jexbot/52.354.77, PMID: 11181715

[ref51] Ragel P. Ródenas R. García-Martín E. Andrés Z. Villalta I. Nieves-Cordones M.. (2015). The CBL-interacting protein kinase CIPK23 regulates HAK5-mediated high-affinity K^+^ uptake in *Arabidopsis* roots. Plant Physiol. 169, 2863–2873. 10.1104/pp.15.01401, PMID: 26474642 PMC4677917

[ref52] Rengel Z. Damon P. M. (2008). Crops and genotypes differ in efficiency of potassium uptake and use. Physiol. Plant. 133, 624–636. 10.1111/j.1399-3054.2008.01079.x, PMID: 18397208

[ref53] Richter C. (1987). NADP^+^ phosphatase: a novel mitochondrial enzyme. Biochem. Biophys. Res. Commun. 146, 253–257. 10.1016/0006-291X(87)90718-2, PMID: 3038107

[ref54] Roberts D. M. Harmon A. C. (1992). Calcium-modulated proteins: targets of intracellular calcium signals in higher plants. Annu. Rev. Plant Biol. 43, 375–414. 10.1146/annurev.pp.43.060192.002111

[ref55] Ruiz J. M. Sanchez E. Garcıa P. C. Lopez-Lefebre L. R. Rivero R. M. Romero L. (2002). Proline metabolism and NAD kinase activity in greenbean plants subjected to cold-shock. Phytochemistry 59, 473–478. 10.1016/S0031-9422(01)00481-2, PMID: 11853741

[ref56] Sanders D. Pelloux J. Brownlee C. Harper J. F. (2002). Calcium at the crossroads of signaling. Plant Cell 14, S401–S417. 10.1105/tpc.002899, PMID: 12045291 PMC151269

[ref57] Shen L. Tian Q. Yang L. Zhang H. Shi Y. Shen Y.. (2020). Phosphatidic acid directly binds with rice potassium channel OsAKT2 to inhibit its activity. Plant J. 102, 649–665. 10.1111/tpj.14731, PMID: 32128922

[ref58] Shin R. Berg R. H. Schachtman D. P. (2005). Reactive oxygen species and root hairs in *Arabidopsis* root response to nitrogen, phosphorus and potassium deficiency. Plant Cell Physiol. 46, 1350–1357. 10.1093/pcp/pci145, PMID: 15946982

[ref59] Shin R. Schachtman D. P. (2004). Hydrogen peroxide mediates plant root cell response to nutrient deprivation. Proc. Natl. Acad. Sci. U. S. A. 101, 8827–8832. 10.1073/pnas.0401707101, PMID: 15173595 PMC423280

[ref60] Snedden W. A. Fromm H. (2001). Calmodulin as a versatile calcium signal transducer in plants. New Phytol. 151, 35–66. 10.1046/j.1469-8137.2001.00154.x, PMID: 33873389

[ref61] Tai L. Li B. B. Nie X. M. Zhang P. P. Hu C. H. Zhang L.. (2019). Calmodulin is the fundamental regulator of NADK-mediated NAD signaling in plants. Front. Plant Sci. 10:681. 10.3389/fpls.2019.00681, PMID: 31275331 PMC6593290

[ref62] Tewari R. K. Kumar P. Sharma P. N. (2007). Oxidative stress and antioxidant responses in young leaves of mulberry plants grown under nitrogen, phosphorus or potassium deficiency. J. Integr. Plant Biol. 49, 313–322. 10.1111/j.1744-7909.2007.00358.x

[ref63] Thoday-Kennedy E. L. Jacobs A. K. Roy S. J. (2015). The role of the CBL-CIPK calcium signalling network in regulating ion transport in response to abiotic stress. Plant Growth Regul. 76, 3–12. 10.1007/s10725-015-0034-1

[ref64] Véry A. A. Davies J. M. (2000). Hyperpolarization-activated calcium channels at the tip of *Arabidopsis* root hairs. Proc. Natl. Acad. Sci. U. S. A. 97, 9801–9806. 10.1073/pnas.160250397, PMID: 10920194 PMC16945

[ref65] Very A. A. Sentenac H. (2003). Molecular mechanisms and regulation of K^+^ transport in higher plants. Annu. Rev. Plant Biol. 54, 575–603. 10.1146/annurev.arplant.54.031902.134831, PMID: 14503004

[ref66] Wang X. P. Chen L. M. Liu W. X. Shen L. K. Wang F. L. Zhou Y.. (2016). AtKC1 and CIPK23 synergistically modulate AKT1-mediated low-potassium stress responses in *Arabidopsis*. Plant Physiol. 170, 2264–2277. 10.1104/pp.15.01493, PMID: 26829980 PMC4825127

[ref67] Wang X. Hao L. Zhu B. Jiang Z. (2018). Plant calcium signaling in response to potassium deficiency. Int. J. Mol. Sci. 19:3456. 10.3390/ijms19113456, PMID: 30400321 PMC6275041

[ref68] Wang Y. Li B. Du M. Eneji A. E. Wang B. Duan L.. (2012). Mechanism of phytohormone involvement in feedback regulation of cotton leaf senescence induced by potassium deficiency. J. Exp. Bot. 63, 5887–5901. 10.1093/jxb/ers238, PMID: 22962680 PMC3467299

[ref69] Wang Y. Wu W. H. (2013). Potassium transport and signaling in higher plants. Annu. Rev. Plant Biol. 64, 451–476. 10.1146/annurev-arplant-050312-120153, PMID: 23330792

[ref70] Wang M. Zheng Q. Shen Q. Guo S. (2013). The critical role of potassium in plant stress response. Int. J. Mol. Sci. 14, 7370–7390. 10.3390/ijms14047370, PMID: 23549270 PMC3645691

[ref71] White P. J. Broadley M. R. (2003). Calcium in plants. Ann. Bot. 92, 487–511. 10.1093/aob/mcg164, PMID: 12933363 PMC4243668

[ref72] Xia Z. Xu Z. Wei Y. Wang M. (2018). Overexpression of the maize sulfite oxidase increases sulfate and GSH levels and enhances drought tolerance in transgenic tobacco. Front. Plant Sci. 9:298. 10.3389/fpls.2018.00298, PMID: 29593762 PMC5857591

[ref73] Xu J. Li H. D. Chen L. Q. Wang Y. Liu L. L. He L.. (2006). A protein kinase, interacting with two calcineurin B-like proteins, regulates K^+^ transporter AKT1 in *Arabidopsis*. Cell 125, 1347–1360. 10.1016/j.cell.2006.06.011, PMID: 16814720

[ref74] Xu Z. Wang M. Guo Z. Zhu X. Xia Z. (2019). Identification of a 119-bp promoter of the maize sulfite oxidase gene (ZmSO) that confers high-level gene expression and ABA or drought inducibility in transgenic plants. Int. J. Mol. Sci. 20:3326. 10.3390/ijms20133326, PMID: 31284569 PMC6651508

[ref75] Xu Y. W. Zou Y. T. Husaini A. M. Zeng J. W. Guan L. L. Liu Q.. (2011). Optimization of potassium for proper growth and physiological response of *Houttuynia cordata* Thunb. Environ. Exp. Bot. 71, 292–297. 10.1016/j.envexpbot.2010.12.015, PMID: 32287506 PMC7112314

[ref76] Yang T. Poovaiah B. W. (2002). Hydrogen peroxide homeostasis: activation of plant catalase by calcium/calmodulin. Proc. Natl. Acad. Sci. U. S. A. 99, 4097–4102. 10.1073/pnas.052564899, PMID: 11891305 PMC122654

[ref77] Yang T. Poovaiah B. W. (2003). Calcium/calmodulin-mediated signal network in plants. Trends Plant Sci. 8, 505–512. 10.1016/j.tplants.2003.09.004, PMID: 14557048

[ref78] Zeng H. Xu L. Singh A. Wang H. Du L. Poovaiah B. W. (2015). Involvement of calmodulin and calmodulin-like proteins in plant responses to abiotic stresses. Front. Plant Sci. 6:600. 10.3389/fpls.2015.00600, PMID: 26322054 PMC4532166

[ref79] Zhang X. Huang G. Bian X. Zhao Q. (2013). Effects of root interaction and nitrogen fertilization on the chlorophyll content, root activity, photosynthetic characteristics of intercropped soybean and microbial quantity in the rhizosphere. Plant Soil Environ. 59, 80–88. 10.17221/613/2012-PSE

[ref80] Zhao L. N. Shen L. K. Zhang W. Z. Zhang W. Wang Y. Wu W. H. (2013). Ca^2+^-dependent protein kinase11 and 24 modulate the activity of the inward rectifying K^+^ channels in *Arabidopsis* pollen tubes. Plant Cell 25, 649–661. 10.1105/tpc.112.103184, PMID: 23449501 PMC3608784

