# Supplementary material for: The Ca2+-CaM Signaling Pathway Mediates Potassium Uptake by Regulating Reactive Oxygen Species Homeostasis in Tobacco Roots Under Low-K+ Stress
Source: Front Plant Sci. 2021 Jun 7;12:658609. doi: 10.3389/fpls.2021.658609 (PMC8216240; doi:10.3389/fpls.2021.658609)
Supplement: Supplementary file 1 [file Table_1.DOCX]

**Table S1.** Primer sequences used in this study.

| Primer name | Sequence information （5’to3’） |
| --- | --- |
| NtSOD-qF | GACGGACCTTAGCAACAGG |
| NtSOD-qR | CTGTAAGTAGTATGCATGTTC |
| NtCAT-qF | TGGATCTCATACTGGTCTCA |
| NtCAT-qR | TTCCATTGTTTCAGTCATTCA |
| NtPOD-qF | CTCCATTTCCATGACTGCTTTG |
| NtPOD-qR | GTTGGGTGGTGAGGTCTTT |
| NKT1-qF  NKT1-qR  NKT2-qF  NKT2-qR  NtKC1-qF  NtKC1-qR  NtKT12-qF  NtKT12-qR  NtHAK1-qF  NtHAK1-qR  NtHAK5-qF  NtHAK5-qR  NtCNGC3-qF  NtCNGC3-qR  NtCNGC10-qF  NtCNGC10-qR  NtActin-qF | TTGCTGGTGATGGTACTTCAG  TACCCGCCCTAGATTAGTCG  GCTCAAGATCATTGCAGGTCAG  CTGAGATCCAAGTTTTGCATGTG  CACTATTGTCATGGCGGATG  TCTTCGGTACATCCGTTTCTG  GCTAGTGTTATGTCAGTGGATG  TCAAACCATGTATGTCATTCCC  CAGGCATGGCGTTTATACT  CCGCGACAATTCCTTCT  ACGGTGGTGGGTGTTTCGGT  ACGAGGATGATCGGGGCGAA  CATGTCTGCAATCCTCAACATT  TAATCCGCTCTCTGAGGTTCTC  GAGGAACTTCTAACATGGGCAC  GTAGAATCGGAACGTGTGTTGA  TGGCATCACACTTTCTACAA |
| NtActin-qR | CAACGGAATCTCTCAGCTCC |
